# Supplementary material for: Are carbon emissions trading and green financial instruments synergistic? -Comprehensive quantitative research based on content analysis
Source: PLoS One. 2024 Mar 7;19(3):e0298601. doi: 10.1371/journal.pone.0298601 (PMC10919601; doi:10.1371/journal.pone.0298601)
Supplement: S1 Data — (ZIP) [file pone.0298601.s001.zip › Data/地级市Z_F工作报告.zip!/╜¡╬≈/╛┼╜¡╩╨/└┤╫╘╣1⁄2╓┌║┼í╛┬φ┐╦╩2╛▌═°í┐_2008.docx]

各位代表：

现在，我代表市人民政府，向大会作工作报告，请予审议，并请各位政协委员和其他列席会议的同志提出意见。

一、二ＯＯ七年工作回顾

２００７年是本届政府工作的开局之年。在省委、省政府和市委的正确领导下，我们深入贯彻落实科学发展观，团结和依靠全市人民，围绕“一个定位”、“四个打造”、“五大战略”，坚持发展为第一要务，民生为第一事务，大力推进沿江开发、项目建设、体制机制创新、新农村建设、民生工程等重点工作，顺利完成了年初确定的各项任务，经济稳健发展，社会和谐进步。

——经济总量扩张。全市实现生产总值５９２亿元，比上年增长１４％；完成财政总收入５７．５亿元，比上年净增１１．７亿元，增长２５．４％，其中地方一般预算收入完成３２．１亿元，增长２３．２％；修水县财政总收入突破５亿元。

——发展速度加快。全市规模以上工业企业达到５８０户，净增８０户；规模以上工业完成增加值１８０亿元，增长２６％，实现利税５２亿元，增长１２０％；全市工业用电量达２４．８亿千瓦时，同比增长２８．１％，是近十年来增幅最大的一年；利税过亿元的企业达到７户，比上年增加４户。

——运行质量提升。财政总收入占ＧＤＰ的比重达到９．７％，比上年提高０．６个百分点，税收占财政总收入的比重达到８４．３％，比上年提高３．７个百分点，三大主体税增加额占增收总量的８７．１％，全年财政保持均衡增长、均衡入库；二、三产业占ＧＤＰ的比重比上年提高３个百分点；民营经济对财政的贡献率比上年提高４．５个百分点。

——发展后劲增强。全市亿元以上重大项目２８４个，比上年净增１７６个，其中１０亿元以上的特大项目１３个。完成城镇固定资产投资３０６亿元，比上年增长３３．８％；年末金融机构存款余额５０１．８亿元，比年初增加５９．８亿元；贷款余额３０８．３亿元，比年初增加３６．８亿元。

——民生有效改善。以民生为重点的社会事业全面发展，投入５．２亿元进行社保、就业、教育、医疗、卫生等民生改善。全市社会消费品零售总额１６３．３亿元，比上年增长１７％。城镇居民人均可支配收入１１５０５元，农民人均纯收入３９３６元，分别比上年净增１９１２元和３８５元。年末城乡居民储蓄存款３１１．３亿元，比年初增加２４．１亿元。城镇和农村人均拥有住房面积３０．２平方米和３７．４平方米，分别增加２．３平方米和０．９平方米。

一年来，全市上下团结奋斗，开拓进取，我们的工作取得了新的明显成就：

（一）不失时机启动沿江开发，在扬优发展上取得突破。开发沿江、做强沿江，实行龙头带动，是九江经济腾飞的必由之路。我们按照省委、省政府把九江建设成为长江沿岸和中部地区重要经济中心城市的全新定位，抓住机遇作出了沿江开发的战略决策，得到了省委、省政府的高度重视和大力支持，提升为全省发展战略。我们秉持“只为成功想办法，不为失败找理由”的理念，以“以港兴城、港城互融、港贸互联、港工互动”为目标，于４月中旬同步启动了规划建设、征地拆迁、投融资平台建设、项目推介和引进，完成了“一核、两轴、六板块”的开发框架总体规划和１０个专项规划，成功引进上海港口集团作为战略投资者参与新的港口和物流园区建设。城西港区作为沿江开发的主板块，提出“决战６００天，建设新港城”，新组建投资公司，融资近６亿元，快速推进疏港大道和商住区建设。沿江各县（市、区）按照功能定位和产业布局，加快项目推进，引进重大项目１３７个，金砂湾板块引进１０亿元以上的大项目４个，园区产业集聚逐步成势。１０月底，省政府发布《江西省九江沿江开发总体规划》，城西港口一期工程奠基及沿江２０个重大项目隆重开工。沿江开发正在成为九江扬优发展的突破口，对外开放的第一形象，招商引资的第一载体，承接产业转移的第一方阵。

（二）强力推进重大项目，发展的能量进一步积蓄。重大项目是经济发展的关键。我们积极开展与经济发达地区、大企业、项目和院校所对接，突出抓好招商引资。九江石化Ⅰ套常减压装置开工建设，原油加工能力将提升到６５０万吨。九江钢厂以新代旧综合改造项目启动，炼钢能力提升到４００万吨。同方江新、江洲、翔升等一批船台项目加速推进，新增造船能力７０万吨。星火有机硅扩改、亚泥三期、汇源三期和凤竹纺织等一批重大项目竣工投产。赛得利二期、Ｌ乳酸、红鹰直升机、大唐氟化工、宝迪灯具和中粮集团油脂加工等重大项目相继开工。全市工业园新增企业７６户。重视矿业经济发展，启动采矿秩序整顿和矿产资源整合行动。积极做好西气东送、川气东输前期准备和九昌樟成品油管线建设。全省首家风电项目在都昌矶山湖破土动工。大力发展循环经济，节能减排初见成效。星火工业园列入国家循环经济试点，九江电厂烟气脱硫、九江石化硫磺回收等环保减排装置投入使用。全市万元ＧＤＰ能耗下降３．８５个百分点，二氧化硫排放下降５．２％，化学需氧量下降２．５％。

（三）新农村建设取得可喜成绩，城乡统筹发展迈出坚实的步伐。去年是我市对农村投入最大、农民群众得实惠最多、农村面貌变化最大的一年。我们坚持统筹城乡发展，始终把“三农”工作放在重中之重。整合１５０多个部门力量，动员社会、企业、部队支持共建新农村。全市按照统一规划，高质量完成了１８０１个村庄的整治，规划有序、凸现田园自然风光的村落随处可见。筹集各类资金４．２７亿元改善农村生产生活条件，完成改路２６００公里，改水６．７万户，改厕５．９万户，新建沼气池２．５万个，积极探索村落社区建设新路径，全市新农村建设跃入全省先进行列。农业产业化稳步发展，“三花烂漫河鲜美，六畜兴旺桑茶香”的产业格局正在形成。全市在稳定传统产业的基础上，新扩九江水梨５万亩，小龙虾、鱼回鱼等特种水产养殖迅速发展，新建成２个国家级水产良种场。新增主营业务收入１０００万元以上的农业龙头企业１０家、亿元以上的３家，培育发展农民专业合作社１３７家，重点扶持４０家。建立万亩、万头（羽）以上的种养基地１３３个，省级无公害农产品基地３０个，培育有机绿色食品品牌６１个，无公害农产品品牌２４个。开放型农业有了新突破，永修宏康、赣台农业示范园等１１５个生产、加工、出口型基地和企业相继落户建成。“万村千乡”工程顺利实施，全市新建农村超市１０００余家，进一步完善了农村流通体系，活跃了农村市场。南方粮食交易市场建立了电子商务交易网络，年交易量达５０亿斤，资金流达８０亿元，成为全国最大的稻米交易市场，九江昔日“四大米市”的辉煌正在重现。综合利用农业开发、扶贫、移民等项目资金，改造中低产田４０．１５万亩，农业综合生产能力明显增强。启动了集体林权制度改革，“山定权、树定根、人定心”的做法得到了中央和省里的充分肯定，受到广大林农的欢迎。县县进入全省林改先进行列。

（四）改革开放不断深化，经济活力进一步显现。高度重视国企改革，建立改制周转金制度，多方筹集改制资金，破解国企改制难题。６０户市属企业启动改制，江西制氧机厂等１１户改制基本完成。靠大联强，成功引进上港集团参与九江港口集团改制，九江港政企分开和港口管理体制改革落实到位。１０户企业政策性破产获得批准。采取盘活存量、打包回购债权等政策，处置债务１６．７４亿元，为下步推进国企改革奠定了基础。深化财政管理，探索现代城市管理与发展的新路子、新模式，进一步简政放权，创新机制，理顺市区两级事权财权，激发了多方面的积极性，九江经济开发区、庐山区和浔阳区活力释放，发展明显提速。金融改革不断深化。全省第一家村镇银行——修水九银村镇银行开业。推进国有资产运营监管体制建设，行政事业单位预算外资金和国有资产清查按期完成，首批６２家市属行政事业单位经营性国有资产完成产权划转，组建市国有资产经营管理中心和担保公司。乡镇综合改革、国有水利工程、种子管理体制改革全面完成，行政事业单位工资制度、机关后勤管理改革稳步实施。鼓励民营经济发展，全民创业日趋活跃。全市私营企业新增２０３９户，个体工商户日均新增８３户。积极搭建政银企合作平台，１４９户中小民营企业与市内八家银行达成贷款协议近５０亿元。

努力提高开放型经济水平，提升对外开放的层次和质量。赴欧洲、新加坡招商，赴香港、上海、佛山推介和浔台经贸洽谈会、中国“五会”等一系列经贸活动，提振了九江的发展形象。对接中央国有大型企业取得成效。全市新批外商投资企业１２７家，实际利用外资３．８亿美元，比上年增长１２％，现汇进资突破１亿美元，比上年增长３１９％。实际利用内资２４０亿元，增长１４．３％。与上海产业对接引进企业８２户，开辟了承接产业转移的新渠道。引进５家总部经济入驻。广泛开展国际交流与合作，积极利用国际高层论坛展示推介九江。新缔结２个国际友好城市，新签２个国际友城意向，成功举办首届国际友城大会。对外贸易大幅增长，外贸出口完成２．５亿美元，增长７１％，九江港集装箱吞吐量达到８．８万标箱，净增１．５万标箱。口岸、海关、检验检疫、海事、边防和外汇管理等进出口通关服务更加快捷便利。侨务、对台事务、驻外机构、商会等在对外开放中发挥了积极作用。

（五）挖掘九江历史文化内涵，推动了文化与旅游的共融发展。广集民智，尊重民意，积极构建城市软实力，确立“融汇九川、敢为人先、勇创实干、追求卓越”的九江城市精神和新的市树（香樟）、市花（荷花），倡引激发全市人民和谐创业的文化力量与热情。成立市文化旅游发展委员会。初步形成了全市文化旅游发展大格局及项目布设规划，组织制定庐山西海、环庐山文化旅游休闲景观带规划。庐山申报国家首批５Ａ级旅游景区一举成功，同时与国旅成功开展战略合作，两个五星级酒店顺利开工。共青西游记文化产业园正式签约，东林大佛等一批文化旅游项目推进顺利，西海温泉、天地温泉竣工开业。全年共引进文化旅游项目签约资金逾百亿元。结合新农村建设，环庐山景观带、庐山西海景观带、昌九沿线环境整治收效明显，新构了旅游城市新特色与大环境。积极推进“五城同创”，成功创建省级卫生城市，省级文明城市通过评审。星子县荣获“中国十佳休闲名县”和“中国最具魅力旅游大县”称号。引入市场机制，成功举办国际龙狮精英赛、世界超级模特大赛、世界旅游小姐大赛、市旅游商品研发大赛、首届金秋饮食文化节，推出了“浔阳鱼席”特色饮食文化品牌。成功举办红色旅游博览会、纪念秋收起义８０周年活动。开展旅游促销，包机游首临九江，游轮游、专列游、自驾游纷至沓来，“农家乐”等特色休闲游不断升温，九江正成为长江中下游休闲度假的热点城市。全年接待国内游客１２１５万人次，增长２０．２％；接待入境游客１５．２万人次，增长２９．９％；旅游总收入８８．３亿元，比上年增长２３．２％。

（六）注重规划引领，着力夯实持续发展的基础。发挥规划在落实科学发展观和经济社会发展中的先导与规制作用，按照“美丽浔阳、怀抱庐山、东临鄱湖、北依长江、西拓港城、南接走廊”的发展构想和“一核、二带、三城、九镇”的城市架构，初步形成《城市发展战略规划》，在全省率先启动生态保护规划。全市县城总体规划修编相继完成，乡镇规划编制率达６０％，村庄规划编制率达７３．７％。全市产业规划、港口岸线利用规划、新农村建设五年规划、矿产资源开发利用规划、商业网点规划等相继完成。土地利用总体规划、沿江高速、庐山西海准高速公路规划全面启动。昌九城际高速铁路顺利开工，武吉高速、铜九铁路（九江段）建设加速推进，福银高速（九江段）改造按期完成。“三年打胜公路建设翻身仗”开局良好，德白线等县、乡公路改造完成，３１６国道改造工程开工。硬化农村公路１５００公里，新增３７５个行政村通油（水泥）路，通畅率达６８％，比上年提高１６个百分点。大力实施“造地增粮富民工程”，新增耕地１．１万亩。争取上级水利建设资金２．７亿元，１７４座病险水库完成除险加固，日元贷款城市防洪工程、鄱湖二期防洪工程、水土保持生态工程、水利血防工程顺利实施，防灾抗灾能力得到增强。注重完善城市功能，滨江东路截污等续建工程顺利完工。供电、供水、供气、通信等公用事业发展，便民服务能力进一步提高。九江、湖口５０万伏输变电工程正式启动。解决了偏远地区农村２２２５户村民用电，实现全市农民用电户户通。开展绿色生态建设七大专项行动，开展昌九绿色走廊建设，实施平原绿化、公路两旁绿化和村落、庭院绿化，城市绿化率达４３％。重点污染源在线监控体系基本建成，城区空气质量达到国家二级标准，饮用水源水质达标率１００％。生态环境进一步改善，荣膺“中国十大宜居城市”。

（七）大力实施民生工程，人民群众得到更多实惠。省政府下达的８大类５２项民生工程８１项考核指标全面达标。同时狠抓养老保险扩面征缴、“零就业”家庭就业帮扶、小额贷款支持创业工作，全市新增城镇就业５．８万人，实现“零就业”家庭１００％就业；养老保险基金征缴总量首次突破６亿；发放创业小额贷款２亿元，比上年增长８０％以上。城镇登记失业率控制在３．６％以内。新增转移农村劳动力就业５万人。城镇职工养老保险参保率近６０％，比上年提高１１个百分点。全年就业培训１０．２万人。第四次提高城市低保标准，为低保户发放物价补贴１０４４万元。农村“五保户”集中供养率达到８０％，比上年提高１６个百分点。城镇居民基本医疗保险全面启动，参保人数达３５万人。新型农村合作医疗扩大到８个县，参合农民２０８万人，参合率达８９％。在全省率先开展“邻近就医同等报销”、“市直医疗单位新农合直补”、“政府购买城市社区公共卫生服务”，完善了困难群众医疗救助制度，扩大了救助范围，提高了救助标准。在全国物价持续走高的形势下，政府积极干预监管，使全市的猪肉、煤气、蔬菜等价格涨幅全年低于全省平均水平。开启蔬菜早市２０处，方便市民。加大城市管理和市场管理力度，市容更加整洁，市场繁荣有序。

（八）努力发展社会事业，保持了社会和谐稳定。鼓励科技创新，加大科技投入，新增高新技术企业３家，专利申请达３００件，实现两年翻番。大力实施名牌战略，新增中国名牌产品１个、中国驰名商标３个，省名牌产品８个。建立义务教育经费保障机制，落实义务教育资金１．９亿元。改建维修校舍１１万平方米，消除危房１５万平方米。新扩建优质高中７所，建成寄宿制农村中小学５５所。扶持社会力量兴办中等职业学校，启动了龙门职教园区建设。开展了周敦颐诞辰９９０周年纪念活动。投入近千万元，广泛开展了“送文化下乡”活动，新增３００个自然村通广播电视，丰富了群众文化生活。拍摄、选送的影片《东京审判》获国家“五个一”工程奖，百集九江方言情景剧《女儿街的婆媳经》，受到市民欢迎。加强疾病防控体系建设，市紧急救援中心和县级疾控中心开始投入使用，争取上级血防补助项目１１个。人口与计划生育工作水平进一步提高，人口自然增长率控制在７．６９‰以内。我市选手参加世界特奥会获１金５银１铜的优异成绩。成功举办了第十届市运会，全民健身掀起热潮。住房公积金覆盖率提高１１个百分点。慈善事业大步推进，募集冠名基金３９００万元、慈善资金３５０万元，在助学、助医、助孤、助残等方面发挥积极作用。普法工作有序推进，应急体系不断健全。“双拥”创建工作荣获全国三连冠。民族宗教、国家安全、国防动员和民兵预备役工作进一步加强。统计、档案、质监、药监、供销、气象、水文、烟草、地方志工作取得新成效，文学艺术、新闻出版、广播电视事业健康发展。妇女、儿童、老年人、残疾人权益得到保障，免费为２０００例白内障患者实施复明手术。开展地质灾害“万村培训”。倾听群众呼声，解决了一大批历史遗留的土地、房产问题。消防安全、安全生产形势保持平稳，各类伤亡事故大幅下降，社会治安综合治理扎实推进，治安形势、信访秩序明显好转，社会保持和谐稳定。

（九）大力推进政府自身建设，政务环境有所改善。富有成效地开展了以“提质、提速、提效”为主题的机关效能建设，政风行风评议扎实推进，机关干部精神面貌焕然一新，机关工作作风不断改进，破解难题的能力进一步提升，工作效率明显提高。完善项目调度、季度分析通报、现场办公等工作推进机制，有效促进了工作的落实。积极推行政务公开、办事公开，行政服务中心集中审批更加便捷有效。启动了公共资源交易中心和便民服务中心建设，电子政务平台投入使用。完善政府新闻发布制度，畅通“民声通道”、“网上信访”，整合市长热线和手机信息平台，开通了政风行风热线直播，中国九江网进入全国地方政府网站４０强。自觉推行依法行政，接受人大法律、工作监督，接受人民政协民主监督和新闻媒体舆论监督。重大问题广泛征求人大代表、政协委员意见，全年提请人大决议案３件，办理人大代表建议２１６件，政协提案５３７件，办结率达１００％。建立健全重大决策调查研究、专家咨询和公众听证制度，注重发挥各民主党派、无党派人士、工商联和人民团体的参政议政作用。深入开展执法监察、审计监督和专项整治，廉政建设不断加强，源头预防治理腐败制度进一步建立，跻身“中部最佳投资（环境）城市”之列。

各位代表，在市十三届人大一次会议上，市政府承诺全年办好十件惠民实事，目前有９件全面兑现，１件正在全力推进。城乡困难群众低保和大病医疗救助全覆盖如期实现，超计划帮助２９６９户“零就业”家庭就业，完成１１４２名残疾人就业。免除城乡义务教育学杂费全面落实，受惠学生数比省下达指标多１．７万人，完成率１０３％，公开招聘２０００名乡村教师顺利完成。廉租房制度进一步完善，８５３户“双困”家庭得到住房保障，建成经济适用房２４．１万平方米，惠及３０８１户中低收入家庭。市区新建公厕２２个，新建垃圾中转站７个，完成农贸市场设施改造４个，另有９座农贸市场建设正在推进。扩建蔬菜基地５０００亩，储备生猪３０００头，城区居民买菜难问题得到一定缓解。解决了１０．４６万人安全饮水。搬迁深山移民９４００人。鹤问湖污水处理工程全面启动。公路客运中心和公交总站主体工程基本完成，正在进行配套设施建设。市民服务中心挂牌运行。九江化工厂搬迁已经启动，正在积极推进。

２００７年，我们立足开好头、起好步，始终注重完善思路与真抓实干、立足当前与筹划长远、对外开放与对内搞活、中心城市与新农村建设、经济发展与社会和谐的统一。具体实践中，一是更加注意把握发展的战略性。把九江置于长江沿岸和中部地区的大格局中去谋划，坚持规划引领，效益优先，追求高起点发展、集约发展。二是更加注意把握发展的开创性。在历届班子打下的良好基础上继承创新，优化思路，完善举措，力求扬优发展、加快发展。三是更加注意把握发展的科学性。既着眼全局，通盘谋划，又抓住牵动全局的重点工作和事关群众的突出问题，以重点突破牵动全面提升，实现协调发展、为民谋发展。四是更加注意把握发展的基础性。立足打基础、利长远，不搞短期行为，着力解决瓶颈制约问题，加强生态保育，促进永续发展。

各位代表，过去的一年，我们坚持以科学发展观为指导，努力适应时代发展的新要求，顺应人民群众的新期盼，迈出了改革开放的新步伐，营造了干事创业的好氛围，开创了和谐稳定的新局面。这是省委、省政府和市委正确领导的结果，凝聚着全市人民的心血和汗水，得益于市人大、市政协的支持和监督，得到了社会各界的关心和帮助。在此，我代表市人民政府，向各位人大代表、政协委员，向奋斗在各条战线的全市广大干部群众，向离退休老同志，向各民主党派、工商联、无党派人士、各人民团体，向驻浔人民解放军和武警官兵，向中央和省驻浔单位，向所有关心、支持和参与九江建设的海内外朋友，表示衷心的感谢并致以崇高的敬意！

在看到成绩的同时，我们也清醒地认识到前进中面临的矛盾和困难，工作中存在的问题和不足。主要是：经济总量和规模仍然偏小，起支撑性作用的骨干项目和企业还不够多，经济结构优化、产业层次升级，尤其是科技创新差距较大；城乡发展不够均衡，县域经济基础仍然薄弱；掌握现代经济发展手段不多，投融资体制机制建设比较滞后；市属国有企业改革任务繁重艰巨；农业龙头企业带动能力不强，现代农业推进力度及新农村建设中支持服务体系建设尚需加强；就业再就业压力仍然较大，物价受大环境影响上涨幅度较大，城乡部分困难群众的生活没有得到根本改善；影响社会和谐稳定因素依然存在；推进科学发展的能力水平有待进一步提高，政务环境有待进一步优化等。对这些问题，我们将认真对待，采取切实有效措施加以解决。

二、二ＯＯ八年工作目标和主要任务

２００８年是实现九江跨越式发展的关键一年，也是建设经济中心城市的重要一年。当前，省委、省政府对九江的发展更加重视和关注，长江沿岸和省内外经济合作更加密切，九江自身的发展优势和发展能量进一步凸现，全市人民加快发展的愿望和期盼更加殷切。今年在国家宏观调控实行从紧的货币政策背景下，发展的压力、挑战也进一步加大。挑战蕴含机遇，机遇稍纵即逝，发展时不我待。新的一年，我们要全面贯彻落实党的十七大精神，深入贯彻落实科学发展观，围绕建设长江沿岸和中部地区重要经济中心城市的目标，拓展改革开放的广度与深度，加快转变经济发展方式，坚持好字优先，突出项目建设，举全市之力推进沿江开发，繁荣发展文化旅游，培育壮大特色产业，统筹区域经济发展，追求绿色生态和谐，加强社会主义新农村建设，大力实施民生工程，努力构建和谐社会，促进全市经济社会又好又快发展。

２００８年经济和社会发展的主要预期目标是：全市生产总值６９０亿元，增长１３％；财政总收入６６．１亿元，增长１５％，力争跨越７０亿元大关，地方一般预算收入３７．２亿元，增长１６％；规模以上工业增加值增长２０％，主营业务收入达到８００亿元；城镇固定资产投资增长２５％；实际利用外资和市外资金分别增长１０％、１５％；外贸进出口总额增长２０％；社会消费品零售总额增长１７％；城镇居民人均可支配收入增长８％，农民人均纯收入增长８％；城镇登记失业率控制在４．５％以内；人口自然增长率控制在７．８‰以内；单位ＧＤＰ能耗下降４％；主要污染物二氧化硫排放下降９．５％，化学需氧量排放下降２．２％；价格总水平涨幅控制在４．５％以内。

为实现上述目标，我们将倍加注重好字优先、加快发展。认清发展不充分这一基本市情，牢牢抓住经济建设这个中心，以科学发展观为指导，敢于发展、善于发展，在做大经济总量的同时，努力提高经济增长的质量和效益。倍加注重深化改革、创新发展。把解放思想置于万事之先，加大改革创新力度，不断破解发展的难题，激活创业创造、科学发展的动力和源泉。倍加注重以人为本、为民谋发展。把各项工作最终落实到为民谋利造福上，尊重群众主体地位，发挥群众首创精神，保障群众各项权益，做到发展为了人民、发展依靠人民、发展成果由人民共享。倍加注重生态文明、和谐发展。牢固树立资源有限、环境无价的理念，注重节能减排，追求环境友好，做到发展经济与保育环境并重，走生产发展、生活富裕、生态良好的文明发展道路。

（一）坚定不移地推进沿江开发战略，举全市之力打造九江新的经济增长极。

按照大格局定位、大产业支撑、大腹地依托、大投入带动、大手笔推进的要求，围绕“一核两轴六板块”的空间架构，着力实施好《江西省九江沿江开发总体规划》和相关专项规划，严守“土地不违规，环保不出问题，防洪不出漏洞”三条底线，矢志不渝地加快推进沿江开发，尽快实现九江在长江沿岸和全省的恢复性占位。

加快沿江基础设施建设。从速构筑沿江两横三纵交通大格局，完成城西港区两个５０００吨级泊位集装箱码头和物流园区建设，启动两个５０００吨级散杂件码头建设。加快推进铜九铁路、昌九城际铁路、城西港区铁路专用线、长江二桥、九瑞高速建设。完成九瑞大道（港区段）扩建工程，加快推进沿江大道改扩建，贯通现代大道、浔阳西路，启动环城高速公路和彭泽沿江高速的前期工作。着力突破城西港区与城区交通瓶颈，构建疏港快速通道。积极做好赛城湖大堤、棉科所大堤的防洪提标，增强沿江园区的排涝能力。加强沿江园区路网、管线、水电气、治污、排污等基础设施建设，高标准建设城西港区环境监控和治污、排污设施，加快湖口、陆家垄、马回岭等输变电站建设，按适度超前的要求建设电网，力争“十一五”期末供电能力达到２００万千瓦，为大工业的发展准备条件。加强口岸基础设施建设，提升口岸综合服务水平。

狠抓沿江重大项目落户。实施核心区带动，沿江产业轴和昌九产业轴联动，加快出口加工区建设，继续把项目建设作为实现沿江率先发展的重要抓手，牢固树立“岸线胜金”、“寸土寸金”的观念，严格执行准入规定，大力提高沿江土地的投资强度和引进项目的产业层级，做到污染项目不进、低端项目不进。各板块按照产业规划的定位，着力引进一批投资规模大、技术含量高、带动能力强、环境友好型的重大项目，力争全年每个板块引进５个２亿元以上项目，２个５亿元以上项目，城西港区力争引进２个１０亿元以上项目。突出抓好已开工的２０个沿江重大项目建设，力促这些项目早日竣工投产。

强化沿江战略实施保障。用足用好政策，积极争资争项，充分发挥财政性资金的撬动作用和市场配置资源的基础性作用，开展多渠道融资。以公司化运作为主体，力争城西港区融资１２亿元，推进４２个项目建设，其它板块新增融资２∽５亿元。建立人才引进机制，着力引进沿江开发急需的管理、物流、融资、招商、技术等方面的人才，千方百计留住人才，用好人才。积极争取上级对沿江开发重大项目用地和环保指标支持，全市项目建设用地指标和环境容量指标向沿江重大项目倾斜。在保障失地农民合法权益的前提下，依法依规、稳步推进项目建设。

（二）加快转变经济发展方式，推动产业经济集聚发展。

发展的支撑在于产业，产业的优势在于集约。强化主导产业规划的引领作用。围绕“六个８００∽１０００”（未来５∽１０年原油加工能力及其油化工、精细化工达到８００∽１０００万吨；船舶、汽车、交通机械及家电机床制造业主营业务收入达到８００∽１０００亿元；钢铁冶炼及加工能力８００∽１０００万吨；电力装机容量力争达到８００∽１０００万千瓦；水泥及建材业生产能力再提升８００∽１０００万吨；纺织工业主营业务收入力争达到８００∽１０００亿元）及“三个３０万吨”（２０１０年玻纤及其制品生产能力、有机硅单体生产能力、有色金属冶炼生产能力分别达到３０万吨）的目标，形成多极支撑的产业经济结构，促使经济发展方式转变。

力推重大产业项目，构筑强有力的产业支撑。抓住九江石化技改，川气、西气东输工程建设的机遇，尽快提升原油加工能力和化肥、化工发展能力；建立船舶制造业研发中心，再引进大型骨干造船企业，促进江洲、同方江新、银星、翔升、东海等造船骨干企业万吨级船台建设；支持昌铃公司新发动机、新车型开发和市场开拓，大力推进汽配和船配产业的发展；实施以新带旧综合利用改造工程，争取金砂湾尽快形成４００万吨钢铁生产能力，加快新上冷轧薄板和镀锌板项目；抓好亚东水泥四期建设和兰丰、三环、鑫山、鼎立公司的技改扩能，提升水泥生产能力；积极推进九江电厂四期“上大压小”项目，加快推进都昌、星子、庐山区等地风电项目开工建设，做好彭泽核电项目的前期工作；力促恒基太阳能热水器、红鹰直升机生产线等项目竣工投产；加紧实施赛得利三期、龙达３０万吨粘胶以及华孚、凤竹、立峰、锦兴、远东等项目，尽快形成化纤、棉纺、面料、服装专业市场齐头并进，链式发展的产业格局。

扶持重点骨干企业，增强衍生和带动能力。围绕提高企业规模经济水平和产业集中度，继续大力扶持九江石化、九江钢厂、九江电厂、香炉山钨业、蓝星星火、蓝星卡博特、赛得利化纤、巨石九江公司、昌铃集团九江分公司、赣北钼业、九江整流器厂、７１３厂等一批骨干企业做大做强。着力培育一批年主营业务收入超２０亿元、超５０亿元、超１００亿元的企业集团。围绕核心企业，着力加强产业上下游、前后道延伸配套，吸引外来企业和民营企业参与产业链分工，进一步拉长、加宽产业链条，形成龙头企业带动、中小企业集群跟进的格局。加强要素保障，促进资源、能源、土地、资金向重点项目、重点企业、支柱产业倾斜，尽快形成一批新的骨干企业。

提高产业科技支撑，实现“九江加工”向“九江制造、创造”跨越。大力运用高新技术改造传统产业，着力引进一批科技含量高、带动能力强的高新技术产业。鼓励发展总部经济，鼓励骨干企业成立研发中心，加强与高等院校、科研院所的有效对接，吸引他们来我市兴办中试基地，鼓励九江本地院所加大研发力度，为我市高新企业的技术孵化创造有利条件。瞄准前沿新兴产业，加强跟进追逐，重视ＩＴ产业，创意、动漫产业，外包服务等新兴业态的发展。进一步整合全市科研资源，加大科技投入，加强科技研发和科技体制创新，努力在产业结构优化、转变经济发展方式上迈开新步伐。

发挥矿产资源优势，培育新的经济增长点。今年要把抓好矿产资源开发秩序整顿、资源整合和精深加工提到重要议事日程。规范矿产资源勘查规划，整顿资源开采秩序，杜绝无序勘探、低端采选和初级原料输出，着力解决采选企业布局不合理、“多小散乱”和低水平重复建设等问题，依法关闭规模效益低、资源浪费和污染严重的小型矿山企业。鼓励深度开发和精深加工，提高采矿、选矿回收率和伴生矿综合利用率，使资源向龙头企业、优势企业、专业化企业集中，实现资源效益最大化，形成以铜、钨、铅锌、硅、石灰石为主的矿业经济区，促使我市资源优势真正转化为产业的优势，进而成为经济优势。

（三）拓展改革开放广度与深度，激活发展的动力和潜能。

坚定不移地推进改革开放，解放思想，创新机制，注入发展的外源动力，激发发展的内在活力，实现经济的持续、活力、快速发展。

全力推进国企改革。着眼于盘活存量，激发活力，实现国有资产保值增值，保障困难企业职工的基本生计，坚定三年完成市属国有企业改制任务的目标不动摇。继续按照“全面启动、分口推进、一企一策、确保稳定”的原则，完成上年已经启动但尚未完成的４２户企业改制，推进并确保启动今年３１户企业改制任务。大力破解资金筹措、债务化解、职工安置、土地运作等难题。一方面优化资源配置，激发企业积极性，走靠大联强之路，积极引进战略投资者，鼓励外资、民营资本参与国企改革。另一方面把稳定作为改制工作的根本，维护职工利益，千方百计安置职工继续就业和转移就业，让职工走出生活困境，让企业重焕生机。加快国有资产经营与监管体制建设，实行国有资产的所有权与经营权分离，建立法人治理机制，确保国有资产有效运营，保值增值。

加快投融资体制创新。进一步改变依赖计划投资、财政拨款的单一投融资体制，推进投资主体多元化。积极搭建投融资平台，坚持“政府推动，市场运作，公司经营”的原则，加快市国有资产经营中心和担保公司建设步伐，组建九江市沿江开发建设投资有限公司、城市建设投资有限公司、公路交通建设投资有限公司。进一步拓宽银行贷款、项目融资、外来投资等融资渠道。完善“政银企”合作对接平台，引导金融部门加大对园区、项目和新农村建设的支持力度，积极吸引各大银行包括外资银行、民营银行和区域性商业银行入驻我市，设立分支机构或代理机构，培育资本市场，增强市内融资能力。积极推动上市融资，探索股权换资本的路子，力争年内庐山旅游集团上市，加快九江商行增资扩股，组建九江银行并推进上市。激活用好民间资本，推进九江长运、共青鸭鸭、财兴卫浴、美庐乳业、博莱药业、民生集团等一批企业筹备上市。简化投资审批程序，降低门槛，鼓励外资、民资参与城市公用事业建设。规范政府投资管理，推进城市建设市场化，探索政府投资项目代建制，提高政府投资效益。

大力发展开放型经济。按照对接项目、对接企业、对接区域、对接院校所的要求，扩大开放领域，优化开放结构，不断提高开放的质量和水平。开展以提高研究项目的决策水平、包装项目的政策水平、推进项目的操作水平为主要内容的“项目建设年”活动。紧盯世界５００强、国内２００强企业和国内外知名科研院所，引进中高端产业和高新技术项目。优化招商理念，变招商引资为招商选资，更加注重招商项目的规模、效益及产业贡献度。围绕支柱产业，精心包装项目，不搞地价、优惠政策的恶性竞争，提高招商实效。改进招商方法，组织专门队伍、专业队伍，锁定目标，敲门招商，以商招商，中介招商，主动应对和积极做好承接东部加工贸易向中西部梯度转移工作。强化招商服务，树立“环境招商”的理念，缩短办事周期，提高工作效率。加强项目调度与服务，继续坚持项目推进五项制度，实施重大项目领导包干责任制，从项目引进落户到建成投产，全过程跟踪服务。进一步建设好电子口岸，优化大通关环境。借助国际友城平台，发挥驻外办事处和商会的桥梁纽带作用，开展各个领域特别是国际经贸交流与合作。力争全年新落户亿元以上项目不少于２００个，其中５亿元以上项目不少于２５个，１０亿元以上项目不少于１０个。

促进民营经济跃升发展。大力培育九江的创业文化，形成民营经济竞相发展的生动局面。进一步加强对民营经济的政策引导和支持，放宽市场准入条件，凡是国家法律法规和行政规章未禁止的即可准入，凡是对外资开放的对民营经济要全面开放，不断拓宽经营领域和发展空间。鼓励和支持民营企业参与国有、集体企业的资产重组，参与公用事业、基础设施和社会事业项目的建设。积极培育成长型中小企业，大力发展现代家庭工业。进一步拓宽中小企业的融资渠道，积极发展面向中小企业的多层次的融资担保，努力营造有利于民营经济发展的市场环境。进一步理顺民营经济服务管理体制，建设好民营企业服务平台，及时解决民营经济发展中遇到的困难和问题，做到政策上更优惠，环境上更宽松，管理上更科学，服务上更优质。

（四）突出特色错位发展，推进县域经济加速崛起。

建立“以工促农、以城带乡”的长效机制，坚持“多予、少取、放活”的方针，加大统筹城乡发展力度，加快县域经济发展。力争今年财政收入实现县县过两亿元，力争过５亿元的县４个，过４亿元的县２个，培育２５个财政收入过千万元的乡镇。

突出新型工业化和新型城镇化，加快县域经济发展。积极探索新型工业化、城镇化道路，把加速园区建设和升级作为主攻方向，在培育特色产业、做大核心企业、完善功能配套、创新管理体制，破解用地、招工、融资“三难”上实现新突破，使工业园区成为县域经济的活力板块，争取全市园区经济进入全省前三位，并培育出５个主营业务收入超百亿元的工业园区。力争每个园区形成１∽２个主导产业，每个县（市、区）至少形成一个销售额５亿元以上的特色产业集群。大力发展乡镇经济，立足本地比较优势，做强特色产业。在区域范围内，实行“异地办厂、原地纳税”，充分调动乡镇招商引资上项目的积极性。鼓励扶持全民创业，大力扶持农村新经济组织尤其是农民专业合作社发展，引导农村能人积极创办企业。积极引导鼓励外出打工人员返乡创业，大力表彰创业先进典型，营造尊重劳动、尊重创业的良好氛围。着力推进新型城镇化，支持重视县域城镇及镇村规划，加快宜居城镇建设尤其是基础设施建设。遵循“工业向园区集中、土地向种养大户集中、人口向城镇集中”的发展规律，推进城乡一体化发展。探索消除城乡二元结构体制障碍，使新型工业化、新型城镇化与解决“三农”问题相互促进，同步推进。

努力创新工作机制，深入推进社会主义新农村建设。按照“走平坦路、喝干净水、上卫生厕、住整洁房、用洁净能源”的要求，继续抓好自然村整治建设，做到力度不减、投入加大、标准不降。在确保完成省级建设点工作任务的同时，科学安排市县共建点，全市今年新增１４３６个自然村建设点。同时，切实把繁荣农村经济、发展新产业、培育新农民、树立新风尚作为新农村建设的重点，不断完善农家书屋等文化设施，推动新农村建设“五新一好”整体推进。认真总结新农村工作经验，狠抓农村经济结构调整，探索和完善新农村建设投入稳定增长机制、帮扶共建机制、协调推进机制、基层群众自治和村落社区管理长效机制，使之更加科学化、系统化、规范化，走出一条具有九江特色的新农村建设路子。

积极发展现代农业，促进农民增收致富。发展现代农业、繁荣农村经济是新农村建设的首要任务。依托优势资源，积极发展绿色农业、生态农业和旅游观光农业，形成“一乡一业”、“一村一品”的产业格局，构建现代高效农业体系。进一步优化农业产业结构和区域布局，提高耕地的利用率和产出率。按照农业产业总体规划，在提升传统优势产业的基础上，集中力量做大做强水产、林果、畜牧、蔬菜、茶叶、蚕桑六大特色主导产业。力争特种水产养殖面积达到５２万亩，完成新扩九江水梨面积８万亩，力争５年内达到５０万亩。围绕主导产业，重点培育一批起点高、规模大、带动力强的龙头企业。依托品牌兴农，努力培育“九江水梨”、“鄱阳湖水产”等一批具有九江特色的优势农产品品牌。加强农业基础设施建设，切实改善以水利为重点的农业生产条件，抓好全市８１座病险水库的除险加固和１至５万亩圩堤整治加固的前期工作。加快农村公路建设，新增３１０个行政村通油（水泥）路，建设砂石路５００公里，水泥路１３００公里，通达率和通畅率分别达到９３％和８０％。新建沼气池２万个。继续做好修水深山区的移民搬迁，启动都昌、德安两县移民搬迁工作。加大农业支持保障体系建设力度，探索建立农业科技创新及推广应用体系、农产品质量安全体系、农业信息和农产品市场体系、农业机械化推广体系、动植物检验防疫体系、农村人才培育等新农村服务支撑体系，努力提高农业的产业化、市场化、信息化、标准化、机械化。整合培训资源，加强对农民农业实用技术和劳动技能培训，提高农民的就业和致富能力。重视发现和培养乡土人才，发挥他们应用和传播先进实用技术的示范带头作用。完善林权制度配套改革，推动林权有序流转。积极推进国有农场税费改革，进一步减轻农工负担，激发农场发展活力。

（五）强力推进文化旅游战略，打造魅力活力宜居城市。

文化旅游既是发展经济的新兴产业，又是弘扬九江人文精神、彰显九江山水生态的载体，依托文化旅游战略推进，努力打造“山水体验之地、创业向往之城、生活品质之都”。

彰显文化旅游特色，提升城市软实力。进一步完善全市文化旅游发展规划，加快旅游资源、资本和服务的全面开放，将“九江十景”和“庐山十景”等文化旅游项目整合包装，推进统一规划，景区合作，整体融资。大力引进有实力的大企业集团开发建设大型文化旅游景区、景点。努力打造庐山国际旅游龙头板块，推动旅游景区合作，探索合作机制，深化合作内容。大力发展休闲、娱乐、服务产业，促进形成旅游大产业格局。加大旅游促销，巩固近程市场，扩大远程市场，在知名的旅游企业和主要客源地设代办处。继续抓好环庐山和庐山西海景区周边的环境整治工作，全面启动庐山西海准高速公路建设和１０５国道改造。加快推进庐山园门（威家）游客集散中心项目建设。开展“十强旅游乡镇”、“十佳特色旅游村”创建和评比活动。打造文化品牌，举办好重大节会。打响山水文化、书院文化、宗教文化、隐逸文化品牌，深度挖掘、保护和传承我市的非物质文化遗产，推进文化与旅游产业的有机结合。进一步宣传和弘扬九江城市精神，深化文化体制机制改革，大力发展文化产业，提高文化创造力。丰富农村偏远地区群众和进城务工人员的精神文化生活。引导人们树立和尊崇社会主义荣辱观，以增强诚信意识为重点，加强社会公德、职业道德、家庭美德、个人品德建设，自觉履行法定义务、社会责任、家庭责任，倡导移风易俗，培育文明风尚。

完善城市功能，提高市民生活品质。以服务生活、美化生活、提升生活为目标，不断加强完善城市基础建设，提升城市品位。突出规划引领，组织对城市远景规划的深入研究和修编，高度重视各类规划的协调与城市资源的优化配置，为城市长远发展打好基础。突出完善城市功能，继续推进环庐山公路绿化二期、公交客运中心二期和公交总站二期工程，建成滨江东路延伸线、琴湖大道、金凤路延伸线、德化东路、两湖活化引水工程、陆家垄路南段。启动城区棚户区危旧房改造、庐山新城、老九瑞路、鹤问湖周边路网、濂溪大道、南山公园、十里广场和城市配套主干管网等城建项目的建设。创新城市功能项目运营机制，对白水明珠等项目进行管理主体招商。突出便民利民，完成１００条背街小巷的改造。完善农贸市场网点和早市点布设，科学布设配电设施，加大供电供水一户一表改造力度，增设一批垃圾处理设施和公厕。突出城市管理，巩固创卫成果，健全城市和社区管理的长效机制，加强市容环境综合整治。切实提高执法队伍素质，在严格执法中充分体现人文关怀，树立和谐城管新形象。完善便民服务中心功能，规范社区物业管理，逐步解决社区办公用房，关爱社区干部。

加快发展现代服务业，增强城市综合竞争力。坚持市场化、产业化、社会化方向，把改造提升传统服务业与发展培育新兴服务业结合起来，不断提高服务业发展水平。大力发展总部经济、会展经济、楼宇经济。制定物流业发展规划，加快引进实力强、服务优的现代物流企业，进一步发展连锁经营、物流配送、电子商务等现代流通方式。完善市场体系，建设一批专业市场和综合市场。抓紧华孚、华南医药等物流项目的对接，力争早日落户开工；尽快启动华东建材物流中心建设，力促华东三期和沃尔玛早日竣工开业。大力发展金融、信息、科技、文化、体育、保险、会计、法律、租赁、中介、社区服务等新兴服务业。鼓励发展专业化的科技研发、技术推广、科技成果转化、工业设计和节能服务业。加快推进公众信息网、社区信息网建设，促进信息资源共享。

（六）全力抓好民生工程，让发展成果广泛惠及民众。

从人民群众最关心、最直接、最现实、最迫切的利益出发，大力实施民生工程。继续坚持新增财力向社会事业、向基层、向群众倾斜，突出解决好教育、就业、养老、低保、医疗、住房、扶贫等关系人民生产生活的重点问题，努力实现城乡困难群众最低生活保障全覆盖，城乡困难群众大病医疗救助全覆盖，城乡义务教育免除学杂费和贫困生资助全覆盖，城乡居民医疗保障制度全覆盖，使全市人民学有所教、劳有所得、病有所医、老有所养、住有所居。

进一步做好就业和再就业工作。坚持把扩大就业放在经济社会发展更加突出位置，建立完善促进就业的长效机制和政策体系，努力实现经济发展与扩大就业良性互动，力争全年城镇新增就业５．３万人，城镇就业率９４％。加大就业培训力度。围绕全年培训１４万人的目标，创新培训方式，扩大培训规模，提高培训质量。进一步整合培训资源，大力推进企业、劳动者、培训机构三位一体的“订单式”、“定向式”培训方式，不断完善政府购买培训和招标培训等机制，开展发放培训券试点，切实帮助劳动者提高就业技能和创业能力。全面落实小额贷款、税费减免、社保补贴、创业指导等各项扶持政策，力争新增小额贷款１亿元。加大就业援助力度，扶持“４０５０”人员再就业５千人，扶持残疾人就业１千人，确保“零就业”家庭安置率１００％，探索建立失地农民就业新途径。鼓励、支持、组织农村劳动力就近就地转移就业，新增农村劳动力转移就业５万人，就近就地转移３万人。逐步实现农村富余劳动力“在新农村居住，到工业园上班”。

扎实推进社会保险和社会救助工作。进一步完善养老保险制度。继续加大养老保险基金征缴力度，以工业园区和非公有制经济组织为重点，全力推进社会养老保险扩面征缴工作，做大养老保险基金总量，努力扩大社会养老保险覆盖面。全年完成养老保险基金征缴７亿元。探索推进被征地农民、农民工参加养老保险工作。使全市养老保险参保率达到６８％。加强基金监管，确保养老基金运行安全和及时足额发放。继续提高城镇企业离退休人员基本养老金标准。进一步完善医疗保障制度。扩大医疗保险覆盖范围，建立多层次、保障功能多样化、资金来源多渠道的医疗保障体系。扩大城乡医疗救助范围，将新增农村低保户全部纳入困难群众医疗救助范围，将城市低保户全部纳入城市大病医疗救助范围，加强对边缘户的救助，取消病种限定，提高救助标准，简化救助手续。继续抓好社区公共卫生服务和村卫生所建设。进一步健全社会救助体系。完善城乡低保制度，新增农村低保５万人，提高低保标准和补差水平。加强城市低保动态管理，继续提高城市低保补助标准。加快敬老院建设力度，力争五保户集中供养率达到８５％。完善城乡临时救助制度，解决人民群众临时性、突发性的生活困难。继续加强猪肉、粮油、蔬菜等食品的价格监控和市场监管，强化价格调节基金征管，增强政府调控价格的能力，保持价格总水平稳定。

（七）推进生态文明建设，促进人与自然互惠共生。

良好的生态环境是九江最优的品牌，推进生态文明建设是我们最大的责任。结合土地利用规划修编，启动完成主体功能区规划工作，大力推进绿色生态建设。

加强资源节约。坚持“惜地如金”、“岸线胜金”，切实提高项目用地的投资强度，努力做到低端产业不进，低档项目不进，确保沿江地区投资强度在１５０万元以上，工业园区在１００万元以上。加强土地盘整清理，对圈而不用、浪费严重的依法依规收回。大力实施“造地增粮富民”工程，全年完成任务１．８万亩，以造补占，持守耕地保有量，为招商引资和项目落户创造条件。发展循环经济和清洁生产，加大节能降耗力度。

加强环境保护。继续深入开展“绿色生态九江建设七个专项整治行动”，健全环境安全应急处理体系和重点污染源在线监控体系。严把环境准入关和环境容量关，严格保护景区、休闲地环境生态，努力建设生态环保型工业园。要按照“集中排污、集中治污、集中监测”的要求建设沿江园区，特别是高起点建设城西港区、金砂湾工业园的环保配套设施，努力把九江经济技术开发区、出口加工区建设成为绿色、环保、生态工业示范园。严格控制和减少二氧化硫、化学需氧量的排放，达到国家控制标准。重视城市垃圾、污水处理，加快设施建设改造，确保鹤问湖污水处理厂年底前投入使用。

加强生态建养。坚持把农村的生态引入城市，把城市的文明导入农村。处理好工业文明与生态文明的关系，开发建设以保护自然生态为前提，发展经济以环境容量为基础，大力发展生态工业、生态农业、生态旅游。保护好山水资源和湿地，抓紧引进和实施鄱阳湖等湿地保护项目，继续开展“修河环保行”活动，加强庐山西海水质保护。加大生态补偿和修复力度，最大限度地减轻矿业生产对生态环境的污染和破坏，实施“百矿披绿”工程，认真组织实施修水、星子两县水土保持防治工程，完成治理面积４０万亩。继续推进平原绿化，农田林网、路旁、滩涂绿化，确保“十一五”期间林木绿化率提高到５４．６６％。加强森林“三防”，确保青山常在、永续利用，确保庐山、星子温泉、庐山西海等旅游景点生态安全。

培育生态理念。倡导生态世界观、生态价值观和生态政绩观，增强企业的环保意识，培育公众的生态伦理，营造尊重自然、热爱自然、善待自然的良好风尚。扩大绿色志愿者队伍，推行义务植树造林和环保义务劳动等公众参与活动，积极推进绿色创建工作，开展“绿色学校、绿色企业、绿色机关、绿色社区、绿色村落、绿色家庭”创建活动，形成见缝插绿、拆墙透绿、楼宇披绿的生态时尚。

（八）推动社会事业全面进步，切实维护社会和谐稳定。

坚持以发展促和谐，以公平求和谐，以稳定保和谐，努力构建和谐社会。

统筹发展各项社会事业。加强人才队伍建设，用三年时间，面向全国高等院校招录１２００名公务员到乡镇、街道培养锻炼。招聘２０００名医护人员充实乡镇卫生院。深化科技管理体制和科研院所改革，优化科技资源配置。围绕主导产业，突出抓好一批重大科技产业化项目。加大“产学研”合作，推进科技——人才——项目对接，促进科技成果转化，加强知识产权保护力度。坚持教育优先，重视支持九江学院、九江职大等大中专院校的建设发展。加大基础教育设施投入，推进中小学危房改造。完善义务教育经费保障机制，积极化解农村“普九”债务。实施留守儿童寄宿制工程，健全困难学生资助制度，保障进城务工人员子女平等就学。加强教师队伍建设，合理优化师生比。进一步规范中小学校长聘任管理。大力提升农村中小学远程教育应用水平，加快普及高中阶段教育，推进高中课程改革，强化素质教育。加强职业教育，加快职业院校建设，结合九江发展，有针对性地培养技能型人才。重视城乡文化事业协调发展，继续推进文化信息资源共享和广播电视村村通工程，启动建设有线电视数字化平移工程，丰富人民群众精神生活。深化公立医院改革，坚持医疗卫生的公益性质，强化公共卫生服务体系建设，继续开展创三甲医院活动，加快中医药事业发展。不断完善卫生监督执法和医疗救治体系，提高农村三级医疗卫生和城乡社区卫生服务能力。强化重大疾病防控，抓好血吸虫病、结核病、艾滋病等重大传染病的防治。大力发展体育事业，积极开展全民健身活动。继续做好“五五”普法。严格执行计划生育基本国策，稳定低生育水平，提高出生人口素质。深入推进老建扶贫工作，改善革命老区和深山移民的生产生活条件。认真做好全国经济普查、人口普查和文物普查工作。继续做好档案、地方志工作，启动第二轮市志修编。

抓好安全生产工作。坚持“安全第一、预防为主、综合治理”的工作方针，强化安全生产管理和监督，有效遏制重特大安全事故，努力实现安全发展。进一步落实安全生产责任制，完善安全生产目标管理考核，加强全市应急信息报送、应急人员培训和应急救援联动指挥系统建设。深化重点行业、领域的整治，突出抓好煤矿、危化品、公共聚集场所、道路交通和水上交通等专项整治，开展安全隐患长年排查并不停顿整改，继续加大监控力度，巩固小煤矿关闭的成果。强化安全宣传教育和培训，加强安全监管队伍建设，加大食品、药品市场的监管力度，切实保障人民群众生命安全和身体健康。

维护社会稳定。加强社会治安综合治理，健全社会治安防控体系，积极打造平安湖区、零案景区、和睦边区、安宁林区、祥和社区。严厉打击各类刑事犯罪和经济犯罪，开展打击偷盗专项整治活动，加强企业、社区治安环境保护，保障人民群众和企业财产安全。重视加强国防动员和民兵预备役工作。饱含真情做好信访工作，认真落实领导信访责任制和信访接待日制度，加大矛盾纠纷排查调处力度，扎实开展重复访、集体访、越级访专项整治活动，更加注重心理疏导和人文关怀，变群众上访为领导下访，变被动堵访为主动走访，切实解决群众的实际困难和关心的问题。

各位代表，为民办实事是人民政府义不容辞的责任。在广集民智、尊重民意的基础上，确定今年做好十件实事：

１、在城区新建２０万平方米的经济适用住房，再解决不少于３０００户中低收入家庭住房困难；完善廉租房制度，对人均７平方米以下的“双困”家庭实行应保尽保；推进县域城镇经济适用房建设和完善廉租住房制度，对人均６平方米以下“双困”家庭实行应保尽保；启动城区棚户区危旧房改造工程；完成７００户农村特困残疾人危房改造。

２、政府新购２０００个就业岗位，其中市本级购买１０００个，解决“零就业”家庭和“４０５０”人员就业。

３、实施农村安全饮水工程，再使１５万农民群众使用自来水；完成深山搬迁移民８０００人。

４、完善城市功能设施，加快体育中心及其附属设施建设，建设博物馆和３个市图书馆区级分馆，启动美术馆、有线电视数字播控中心、文化演艺中心、第二生活垃圾处理场和大型游乐设施建设。

５、切实抓好“菜篮子”工程，新扩建１００００亩蔬菜基地；每个县建好一个食品检测检疫站。

６、整合交通资源，完善城区公交和城郊线站体系，添置环保型公交车６８辆；完成１５个乡镇汽车站和２００个农村候车亭建设；再完成４５０个自然村通广播电视。

７、整合城区教育卫生资源，完善城区义务教育和卫生网点布局，浔阳区、庐山区、开发区各新建一所义务教育学校；在开发区、庐山区按三级标准各新建一所综合性医院，支持６６０个社区、村卫生服务所（站）建设，免费培训１８００名村卫生所人员。

８、关爱老年人、残疾人，实施九江市老干部休养所改造；每个县（市、区）建成一个标准较高的老年公寓；再免费为２０００名白内障患者实施复明手术。

９、扩大城区管道天然气供气范围，完善城区天然气管网，新增管网１００公里、用户１万户。

１０、启动十里（濂溪）河改造工程，完成塔岭南北路、庾亮南北路人行通道设施建设。

三、加强政府自身建设

按照党的十七大提出建设服务型政府的新要求，以开明开放的工作理念、创新求进的工作思路、争先进位的工作意识、有效融通的工作举措、止于至善的工作追求、高效廉洁的工作纪律，立志有为，履职尽责，为民服务，加强政府自身建设，进一步树立“亲民为民、创新求进、诚信务实、高效廉洁”新形象。

坚持开拓创新，不断提高贯彻落实科学发展观的能力和水平。各级政府和全体工作人员要深入学习贯彻党的十七大精神，贯彻落实科学发展观，牢固树立正确的政绩观，始终抓住发展这个第一要务、民生第一事务，增强发展的责任意识、创新意识和落实意识。深刻领会和准确把握中央和省、市的方针政策、决策部署，创造性地开展工作。遵循客观规律，既尽力而为，又量力而行，不搞不切实际、不顾民力、不计长远的“形象工程”，着眼于解决改革发展稳定中的实际问题，掌握运用现代经济手段，形成谋划工作的思路、促进工作的措施、推展工作的方法、破解难题的路径、领导工作的本领。掌握统筹兼顾的根本方法，既突出重点，又兼顾其它，坚持从全局出发，集成思考，系统设计，协调好各种利益关系，发挥各方面的积极性，实现局部与全局、局部与局部最优互动；深入调查研究，求教于群众，问计于基层，借智于专家，在集思广益中做出科学决策。重大决策尤其是关涉群众切身利益的事情，都要集体决策、慎重决策和阳光操作，重大问题注重听取人大、政协意见，报党委审定，并接受多方监督。切实提高决策水平和实施效果，倡导操作到位是真本领，落实到位是硬功夫。坚决防止以会议落实会议、以文件落实文件，定下来的事情就雷厉风行、一抓到底，部署了的工作就紧盯不放、跟踪问效。改变作风，狠抓落实，大力提倡“一线工作法”，沉到基层，面向群众，直面矛盾，破解难题，不断取得又好又快发展的实际成效。

切实履行职责，进一步优化政务环境。秉持“企业创造财富，政府创造环境”理念，突出“为发展、为基层、为企业、为群众”服务，以提高服务质量为主题，继续深入推进以“提质、提速、提效”为重点的机关效能建设。增强大局观念和服务意识，解放思想，融通办事，只要是有利于发展的事情，就要视为己任，竭诚服务。对基层和客商的诉求，必须积极主动地回应和落实。对群众和企业的实际困难，要设身处地、带着感情设法解决。进一步提高机关办事效率，减化办事程序，坚决消除工作中的“中梗阻”现象。破除狭隘的部门利益观，不允许遇到利益“抢篮球”、碰到矛盾“传排球”、推诿责任“踢足球”的现象发生。继续深入开展政风行风评议工作，再添措施，强化力度，整治环境。对损害破坏经济发展环境的人和事，坚决查处，决不手软，努力营造同心、同向、同力服务发展的浓厚氛围。

突出勤政为民，倾心竭力办好利民之事。牢记“人民政府为人民”的宗旨，勤勉理政、执政为民。自觉接受人大及其常委会和人大代表的监督，加大与人民政协的联系和协商，认真办理人大代表议案、建议和政协委员提案，严格按照法定的权限和程序履行职责，做到依法行政、民主行政、科学行政。以民生为第一事务，做到重大决策依民而定，工作措施应民而出，工程设施为民而建，安危冷暖唯民所系，改革发展成果由民共享。注重履行社会管理和公共服务职能。建立健全群众反映诉求的通畅渠道和回应机制，关注群众日常生活，从解决群众“最盼”、“最急”的问题入手，体现人民政府的真情和作为。在城乡建设和社会管理方面，更多地体现人文关怀和心理疏导。

加强廉政建设，营造风清气正的施政环境。注重廉政和法制教育，引导政府工作人员筑牢思想道德防线，树立法治观念，依法办事，按规矩办事，做到敬畏事业，增强工作责任心；敬畏权力，用好人民赋予的公权；敬畏群众，积极为百姓排忧解难。推行“阳光政务”，深入实施政务公开、厂务公开、村务公开、办事公开，扩大群众有序参与。认真履行廉政建设责任制，健全重大事项报告制度、质询制度、问责制度和民主评议制度。规范权力运行，加强对土地使用权出让、建设工程招投标、政府采购、产权交易等重点环节的监督。加大对专项资金管理使用的监督，加快完善市公共资源交易中心建设，推进源头预防和治理腐败的制度建设。做到行政权力运用到哪里，职能监督就到那里；公共服务提供到哪里，绩效评估就到那里，努力从制度上防止权力寻租，铲除滋生腐败的土壤。发扬艰苦奋斗、勤俭节约的传统，从领导干部和机关做起，勤俭办事业，反对大手大脚、铺张浪费。深入开展执法监察、效能监察、纠风治乱工作，切实维护广大人民群众的根本利益。

各位代表，我们正站在新的历史起点上，建设“中心城市”和全面建设小康社会的宏伟目标激励着我们，人民的重托千钧在肩，发展的使命任重道远，我们必须只争朝夕、奋发进取，努力保持昂扬向上、追求卓越的精神状态，把全部心智倾注到干事业上，把全部精力集中到办实事上，凝心聚力谋发展，和衷共济兴九江。让我们高举中国特色社会主义伟大旗帜，全面贯彻党的十七大精神，贯彻落实科学发展观，在省委、省政府和市委的坚强领导下，团结带领全市人民，振奋精神，顽强拼搏，为建设长江沿岸和中部地区重要的经济中心城市而努力奋斗！
